# Epidemiological Trends, Transmission Dynamics, and Mortality Patterns of HIV/AIDS in South Korea, 1985–2024: A Comprehensive Regression and Time Series Analysis with Projections to 2028

**DOI:** 10.3390/v18070784

**Published:** 2026-07-17

**Authors:** Hyeran Jung, Minsun Jung

**Affiliations:** Department of Pathology, Yonsei University College of Medicine, 50-1 Yonsei-ro, Seodaemun-gu, Seoul 03722, Republic of Korea; phdgrace@yuhs.ac

**Keywords:** HIV, AIDS, epidemiology, South Korea, regression analysis, time series forecasting, male-to-male sexual transmission, MSM, mortality, KDCA, KOSIS, antiretroviral therapy, PrEP

## Abstract

**Background:** South Korea has documented HIV cases since 1985; however, comprehensive longitudinal analyses integrating incidence, mortality, transmission dynamics, and immunological staging across the full surveillance period remain limited. This study aimed to characterize epidemiological trends from 1985 to 2024 and project future trajectories through 2028. **Methods:** We analyzed nationally reported aggregate HIV/AIDS surveillance data from the Korea Disease Control and Prevention Agency (KDCA) via KOSIS (1985–2024). Annual HIV incidence, AIDS case notifications, HIV-related mortality, transmission route distributions, CD4+ T-cell counts at diagnosis, and cumulative prevalent cases stratified by sex were examined. Statistical methods included simple and multiple linear regression, Pearson and Spearman correlation, hierarchical regression analysis, and time series forecasting using Holt–Winters exponential smoothing and ARIMA (AutoRegressive Integrated Moving Average) modeling. Missing data in transmission route classification (29.6% non-response in 2024) were addressed through sensitivity analysis and explicit discussion of potential misclassification bias. **Results:** HIV annual incidence increased significantly from one case in 1985 to a peak of 1081 in 2014 (slope = +39.25 cases/year, R^2^ = 0.912, *p* < 0.001), followed by a significant decline to 714 cases in 2024 (slope = −38.76 cases/year, R^2^ = 0.879, *p* < 0.001). A strong positive correlation was observed between HIV and AIDS incidence (Pearson r = 0.627, *p* = 0.017; Spearman ρ = 0.785, *p* < 0.001). The AIDS/HIV notification ratio declined significantly from 0.307 in 2011 to 0.196 in 2024 (slope = −0.006/year, R^2^ = 0.421, *p* = 0.012). The male-to-female notification ratio increased from 0.8:1 in 1987 to 22.0:1 in 2024 (R^2^ = 0.600, *p* < 0.001). Among reported sexual transmissions, the proportion attributable to MSM increased from 0% in 1985 to 63.7% in 2024 (slope = +1.30%/year, R^2^ = 0.804, *p* < 0.001). The proportional mortality rate declined significantly from 2.31% in 2011 to 0.93% in 2024 (slope = −0.105%/year, R^2^ = 0.821, *p* < 0.001). Time series forecasting projected continued decline to approximately 666 new HIV cases and 105 AIDS notifications annually by 2028. **Conclusions:** The South Korean HIV epidemic has undergone a profound epidemiological transition—from heterosexual-predominant early growth through a 2014 peak toward a declining, MSM-concentrated trajectory—with mortality progressively decoupled from incidence through expanded antiretroviral therapy coverage. Targeted MSM-focused prevention, expansion of PrEP access, and sustained ART scale-up are essential to achieving national HIV elimination targets.

## 1. Introduction

Human immunodeficiency virus (HIV) infection remains a major global public health challenge. According to the 2024 UNAIDS report, approximately 39.9 million people are living with HIV worldwide, with 1.3 million new infections and 630,000 AIDS-related deaths recorded in 2023 [1]. In the Asia-Pacific region, new HIV infections and AIDS-related deaths have shown divergent trajectories compared with sub-Saharan Africa, with some countries achieving notable reductions in transmission while others continue to face persistent epidemic growth [2]. South Korea, a high-income country with a well-developed public health infrastructure, has maintained relatively low HIV prevalence since the first documented domestic case in 1985; however, the epidemic has undergone substantial demographic and behavioral shifts across four decades of continuous national surveillance [3].

Since the enactment of the Acquired Immune Deficiency Syndrome (AIDS) Prevention Act in 1987, South Korea has operated a mandatory national notification system covering all newly diagnosed HIV infections among Korean nationals. This system has generated an uninterrupted surveillance dataset spanning four decades, offering a unique opportunity to examine long-term secular trends in HIV incidence, AIDS progression, mortality, and transmission dynamics within a single national framework [4]. Despite this rich surveillance infrastructure, comprehensive epidemiological analyses integrating multi-dimensional longitudinal data—including sex-stratified trends, transmission route evolution, immunological staging at diagnosis, and rigorous statistical modeling—across the full 1985–2024 period have not previously been reported.

Understanding these trends is critical for several reasons. First, the demographic profile of newly diagnosed HIV-positive individuals in South Korea has progressively shifted toward younger males engaging in male-to-male sexual contact (MSM), reflecting broader behavioral and social changes [5,6]. Second, the introduction of and expanded access to combination antiretroviral therapy (cART) have dramatically altered the natural history of HIV infection, with direct implications for AIDS case notification rates and HIV-attributable mortality [7,8]. Third, the expansion of pre-exposure prophylaxis (PrEP) availability in South Korea since 2019 and the ongoing role of post-exposure prophylaxis (PEP) programs represent important programmatic interventions that may influence future incidence trajectories, yet their population-level impact has not been quantified in a comprehensive longitudinal analysis. Fourth, projection of future HIV incidence is essential for planning HIV testing infrastructure, clinical care capacity, and evidence-based prevention programs aligned with the UNAIDS 95–95–95 targets [9].

The present study addresses these gaps by conducting a comprehensive epidemiological analysis of Korean HIV/AIDS national surveillance data from 1985 to 2024. We employed multiple statistical approaches—including simple and multiple linear regression, Pearson and Spearman correlation analysis, hierarchical regression, and time series forecasting—to characterize long-term trends in HIV incidence, AIDS notifications, mortality, transmission route evolution, CD4+ T-cell counts at diagnosis, and sex-specific epidemiology. Annual HIV incidence and AIDS notifications are further projected through 2028 using validated time series methods.

## 2. Materials and Methods

### 2.1. Data Sources

This study analyzed aggregate national HIV/AIDS surveillance data published through the Korean Statistical Information Service (KOSIS; https://kosis.kr), compiled by the KDCA from the annual HIV/AIDS Notification Status Report. The KOSIS data represent comprehensive national surveillance figures covering all HIV-positive Korean nationals reported under the mandatory notification provisions of the AIDS Prevention Act. All data are publicly available as pre-aggregated annual national statistics; individual-level data were neither accessed nor analyzed.

Seven categories of datasets were obtained: (1) annual new HIV infection notifications (1985–2024), stratified by sex; (2) HIV incidence rates per 100,000 population and AIDS case notifications with corresponding rates (2011–2024); (3) HIV-related annual deaths (2011–2024), stratified by sex; (4) cumulative HIV-infected individuals and estimated living HIV-positive patients (2011–2024); (5) HIV transmission route distributions by sex (1985–2024); (6) CD4+ T-cell counts at diagnosis, stratified by category and sex (1985–2024); and (7) age-stratified HIV notifications (2008–2024).

### 2.2. Statistical Analysis

All analyses were performed using Python (version 3.11) using SciPy (v1.11), statsmodels (v0.14), pandas (v2.0), and NumPy (v1.25). Statistical significance was defined as α = 0.05 (two-tailed). Descriptive statistics were calculated for annual HIV incidence, including mean, median, standard deviation (SD), minimum, maximum, skewness, and kurtosis.

Simple linear regression was applied to characterize secular trends in: (a) overall annual HIV incidence versus calendar year (1985–2024); (b) sex-stratified HIV incidence; (c) AIDS notifications (2011–2024); (d) annual HIV-related deaths; and (e) the proportional mortality rate (annual deaths as a percentage of cumulative living HIV patients). Two-phase segmented regression was applied to the incidence trend, with a structural breakpoint at 2014 (peak incidence year), yielding separate regression models for the ascending phase (1985–2014) and the descending phase (2014–2024).

Pearson (r) and Spearman (ρ) correlation coefficients were computed for bivariate associations, including: HIV incidence versus AIDS notifications; HIV incidence versus annual mortality; AIDS notifications versus annual mortality; and the male-to-female (M:F) notification ratio trend. Multiple linear regression was performed to assess the joint predictive value of HIV incidence and AIDS notifications on annual deaths. Hierarchical multiple regression was conducted in three sequential blocks: Block 1 (calendar year alone), Block 2 (calendar year + HIV incidence rate), and Block 3 (calendar year + HIV incidence rate + AIDS rate), with ΔR^2^ assessed at each step.

The annual proportion of newly diagnosed individuals with CD4+ T-cell counts below 200 cells/μL was calculated and regressed against calendar year to assess immunological staging trends. Given that the “test not performed” category comprised a substantial proportion of annual totals (particularly post-2017), the CD4+ proportion was computed relative to total annual notifications, acknowledging that the true proportion among tested individuals would be higher. This is discussed explicitly as a potential bias in Section 4.

Regarding missing data in transmission route classification, a “no response” category comprised 29.6% of all cases in 2024 (211 of 714 cases). The potential impact of this non-response on the reported MSM proportion (63.7% of reported sexual transmissions) is addressed through a sensitivity analysis assuming three scenarios: (1) all non-respondents are MSM (upper bound), (2) all non-respondents are heterosexual (lower bound), and (3) non-respondents follow the same distribution as respondents (base case).

Time series forecasting was conducted for annual HIV incidence and AIDS notifications using Holt–Winters double exponential smoothing with a damped additive trend (optimized smoothing parameters). Annual HIV-related mortality was forecast using an ARIMA(1, 1, 0) model. Prediction intervals for the Holt–Winters model represent approximate 95% confidence bounds estimated as ±8% of the point forecast for HIV and ±15% for AIDS notifications, based on model residual standard errors. Forecasts were generated for 2025–2028.

### 2.3. Ethics Statement

This study was approved by the Institutional Review Board of Chungnam National University (IRB No. 202601-SB-020-01; confirmation date: 15 March 2026). Patient consent was waived because this study used de-identified aggregate national statistics published through KOSIS and did not involve direct contact with human participants. All analyses were performed exclusively on pre-aggregated, publicly available annual national statistics.

## 3. Results

### 3.1. Overall HIV Incidence Trend (1985–2024)

**Figure 1:**
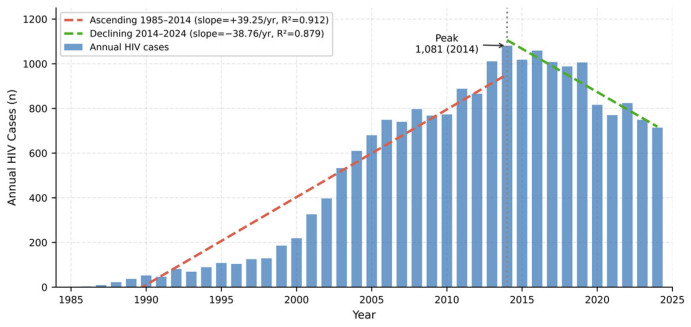
Annual HIV incidence in South Korea, 1985–2024. Bars represent annual new case counts. Dashed lines show segmented linear regression fits for the ascending phase (1985–2014, red; slope = +39.25/year, R^2^ = 0.912) and declining phase (2014–2024, green; slope = −38.76/year, R^2^ = 0.879). The 2014 peak (*n* = 1081) is annotated.

A total of 20,451 cumulative HIV cases were reported in South Korea from 1985 through the end of 2024 (Table 1). Annual new HIV infections increased from one case in 1985 to a peak of 1081 in 2014, followed by a progressive decline to 714 cases in 2024. Overall linear regression across the full 40-year period demonstrated a highly significant positive trend (slope = 30.33 cases/year, R^2^ = 0.828, *p* < 0.001; Figure 1). Descriptive statistics revealed a mean annual incidence of 511.3 cases (SD = 384.7), median 645.0, minimum 1, and maximum 1081.

Two-phase segmented regression identified two statistically distinct epidemiological periods (Table 2). During the ascending phase (1985–2014), the slope was +39.25 cases/year (R^2^ = 0.912, *p* < 0.001), indicating rapid, sustained epidemic growth. The post-peak descending phase (2014–2024) exhibited a slope of −38.76 cases/year (R^2^ = 0.879, *p* < 0.001), representing a statistically significant and accelerating reversal. By 2024, annual incidence had declined 34.0% from the 2014 peak.

### 3.2. HIV and AIDS Notifications and Their Relationship (2011–2024)

**Figure 2:**
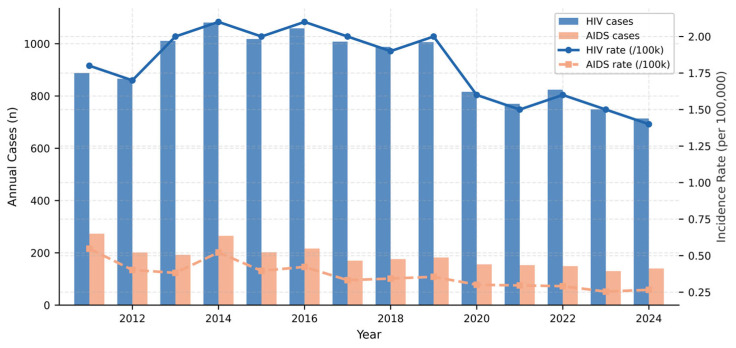
Annual HIV and AIDS notifications and incidence rates, South Korea, 2011–2024. Grouped bars show HIV new cases (blue) and AIDS notifications (orange) on the left axis; lines show corresponding incidence rates per 100,000 population on the right axis.

HIV notifications are expressed as both case counts and incidence rates to facilitate comparison; these terms are used interchangeably throughout, with “HIV notifications” referring to newly reported cases under the mandatory surveillance system. HIV incidence rates ranged from 1.4 to 2.1 per 100,000 population during 2011–2024, peaking in 2014 and 2016 and declining to 1.4 per 100,000 in 2024 (Table 1, Figure 2). AIDS case notifications ranged from 130 (2023) to 273 (2011), with rates of 0.254 to 0.548 per 100,000. AIDS notifications exhibited a highly significant declining linear trend (slope = −8.81 cases/year, R^2^ = 0.729, *p* < 0.001).

The AIDS/HIV notification ratio decreased from 0.307 in 2011 to 0.196 in 2024, representing a significant downward trend (slope = −0.006/year, R^2^ = 0.421, *p* = 0.012), indicating a declining proportion of individuals presenting with advanced HIV disease at notification. Pearson correlation between annual HIV notifications and AIDS notifications was significant (r = 0.627, *p* = 0.017), as was the Spearman rank correlation (ρ = 0.785, *p* < 0.001), confirming a robust positive association.

### 3.3. HIV-Related Mortality Trends (2011–2024)

**Figure 3:**
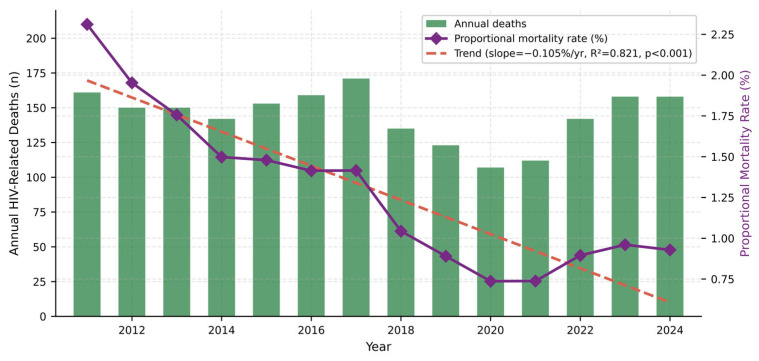
Annual HIV-related deaths (bars) and proportional mortality rate (deaths per cumulative living HIV patients × 100%; purple diamonds), South Korea, 2011–2024. Dashed line shows fitted linear regression (slope = −0.105%/year, R^2^ = 0.821, *p* < 0.001).

Annual HIV-related deaths ranged from 107 (2020) to 171 (2017). Simple linear regression of absolute death counts on calendar year did not reach statistical significance (slope = −1.33 deaths/year, R^2^ = 0.086, *p* = 0.309). However, the proportional mortality rate declined markedly from 2.31% in 2011 to 0.93% in 2024 (slope = −0.105%/year, R^2^ = 0.821, *p* < 0.001; Figure 3), reflecting the growing denominator of individuals living with HIV under effective ART.

Pearson correlation between annual HIV notifications and deaths was non-significant (r = 0.191, *p* = 0.511), as was the correlation between AIDS notifications and deaths (r = 0.243, *p* = 0.403). Multiple regression with HIV notifications and AIDS notifications as predictors of annual deaths yielded a non-significant model (R^2^ = 0.062, adjusted R^2^ = −0.109, F(2, 11) = 0.361, *p* = 0.705). Hierarchical regression (Block 1: calendar year, R^2^ = 0.086; Block 2: + HIV rate, R^2^ = 0.086, ΔR^2^ = 0.000; Block 3: + AIDS rate, R^2^ = 0.086, ΔR^2^ = 0.000) demonstrated that neither notification rate improved prediction of deaths beyond calendar year alone.

### 3.4. Sex-Stratified Trends

**Figure 4:**
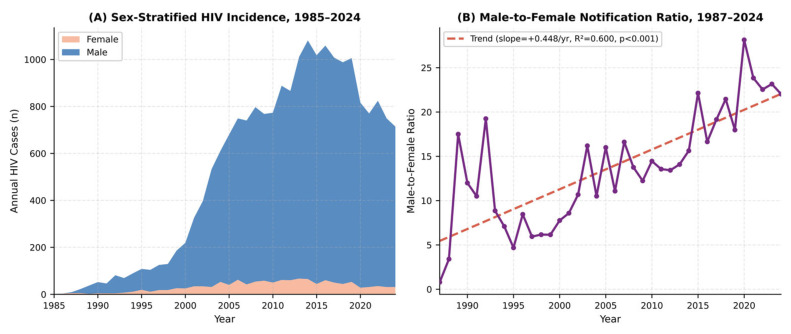
Sex-stratified HIV epidemiology, South Korea, 1985–2024. Panel (**A**): stacked area chart of annual male (blue) and female (orange) HIV notifications. Panel (**B**): male-to-female notification ratio, 1987–2024, with fitted linear regression line (slope = +0.448/year, R^2^ = 0.600, *p* < 0.001).

Males consistently dominated newly reported HIV infections throughout the study period. Male HIV notifications increased significantly (slope = +28.98/year, R^2^ = 0.836, *p* < 0.001), while female notifications increased at a far slower rate (slope = +1.35/year, R^2^ = 0.552, *p* < 0.001; Figure 4). In 2024, males accounted for 683 of 714 new cases (95.7%). The M:F notification ratio increased from 0.8:1 in 1987 to 22.0:1 in 2024 (R^2^ = 0.600, *p* < 0.001). Cumulative living male HIV patients reached 15,975 in 2024, versus 1040 female patients.

### 3.5. Transmission Route Dynamics

**Figure 5:**
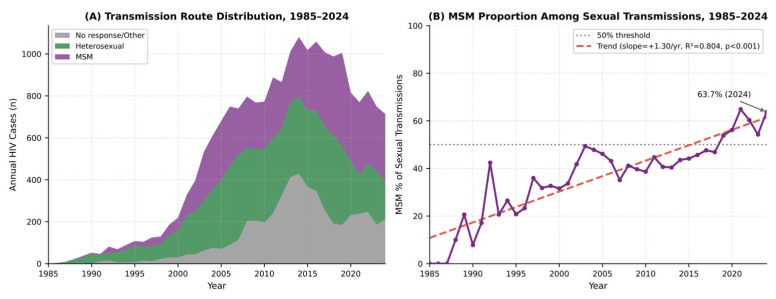
Transmission route dynamics, South Korea, 1985–2024. Panel (**A**): stacked-area counts of MSM (purple), heterosexual (teal), and no response/other (grey) transmissions. Panel (**B**): proportion of MSM among reported sexual transmissions, with linear regression trend (slope = +1.30%/year, R^2^ = 0.804) and the 50% threshold (dotted line) and 2024 value (63.7%) annotated.

Among all reported sexual transmissions, the proportion attributable to MSM increased from 0% in 1985 to 63.7% in 2024, with a highly significant positive linear trend (slope = +1.30%/year, R^2^ = 0.804, *p* < 0.001; Figure 5). Heterosexual transmissions, while numerically stable, constituted a progressively declining share of reported sexual routes (182 cases in 2024).

A “no response” category accounted for 211 of 714 cases (29.6%) in 2024. To assess the potential impact of these missing data on the reported MSM proportion, a sensitivity analysis was conducted under three scenarios: (1) if all non-respondents were MSM, the MSM proportion would rise to approximately 75.4% of total cases; (2) if all were heterosexual, the heterosexual proportion would rise to 55.1%; (3) under the base-case assumption (non-respondents follow the same distribution as respondents), the reported 63.7% MSM proportion among sexual transmissions remains the best available estimate. While the direction of the epidemic shift toward MSM-concentrated transmission is robust across all scenarios, the exact magnitude is subject to non-response bias. Stigma associated with MSM identity may plausibly increase non-response in this group, suggesting the true MSM proportion may be an underestimate.

### 3.6. CD4+ T-Cell Count at Diagnosis

The proportion of newly diagnosed individuals presenting with severe immunosuppression (CD4+ < 200 cells/μL) showed a significant positive trend from 1990 to 2024 (slope = +0.0044/year, R^2^ = 0.336, *p* < 0.001), calculated relative to total annual notifications. A large “test not performed” category persisted throughout the study period, particularly after 2017, reflecting systematic gaps in immunological staging at the time of notification. Because CD4+ testing was not performed in a substantial proportion of cases, the proportion of those tested who had CD4+ < 200 cells/μL would be higher than the overall estimate reported here. This ascertainment bias—whereby sicker patients presenting with symptoms may be more likely to have CD4+ tested—could paradoxically inflate the observed proportion of late-stage diagnoses among tested individuals. The observed significant positive trend in late diagnosis thus reflects a genuine public health challenge but may be partially attributable to selective testing patterns rather than a true population-level worsening of late presentation.

### 3.7. Time Series Forecast (2025–2028)

**Figure 6:**
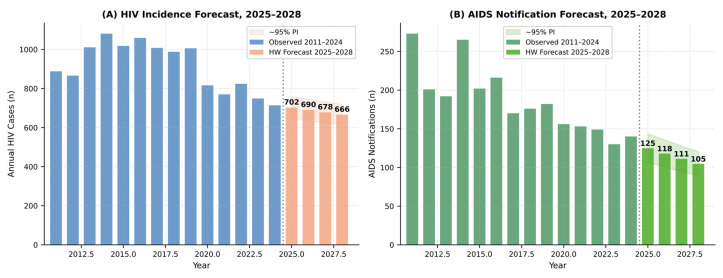
Time series forecast of annual HIV incidence (**A**) and AIDS notifications (**B**), South Korea, 2025–2028. Observed data (2011–2024) are shown as blue/teal bars; projected values as orange/green bars with approximate 95% prediction intervals (shading). Holt–Winters damped additive trend model was applied.

**Figure 7:**
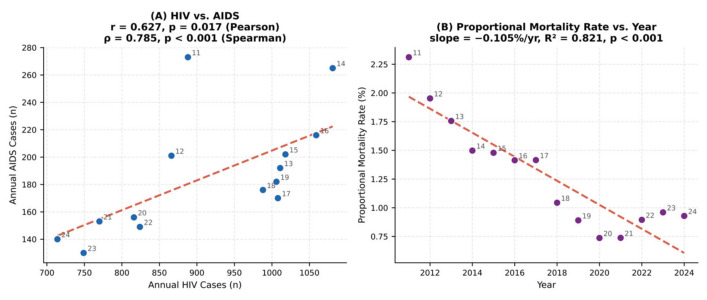
Correlation and regression analyses, South Korea, 2011–2024. Panel (**A**): scatter plot of annual HIV notifications versus AIDS notifications, with Pearson r = 0.627 (*p* = 0.017) and Spearman ρ = 0.785 (*p* < 0.001). Panel (**B**): proportional mortality rate versus calendar year with linear regression line (R^2^ = 0.821, *p* < 0.001). Data points are labeled by year.

Holt–Winters damped trend exponential smoothing projected continued gradual decline in annual HIV notifications: 702 in 2025, 690 in 2026, 678 in 2027, and 666 in 2028 (Figure 6, Table 3). Approximate 95% prediction intervals (PI) are presented in Figure 6 for transparency regarding forecast uncertainty. AIDS notifications were projected to decline from 125 (2025) to 105 cases (2028). ARIMA(1, 1, 0) modeling yielded a stable forecast of approximately 158 deaths per year through 2028, consistent with the observed plateau in absolute deaths while the proportional mortality rate continues its significant decline. The underlying correlation and regression analyses are summarized in Figure 7.

## 4. Discussion

This study provides the most comprehensive multi-dimensional statistical analysis of South Korea’s HIV/AIDS epidemic to date, encompassing four decades of national surveillance data. Our principal findings demonstrate a biphasic epidemic trajectory—marked acceleration from 1985 to 2014 followed by statistically significant deceleration—alongside a profound demographic shift toward MSM-concentrated transmission, a significant decline in the AIDS/HIV notification ratio consistent with earlier diagnosis, and markedly falling proportional mortality rates attributable to expanded ART access.

The identification of 2014 as the epidemic inflection point is epidemiologically significant. This peak may be associated with the convergence of expanded HIV testing programs, intensified MSM-targeted outreach, and the global shift toward earlier ART initiation following the 2013 World Health Organization (WHO) guidelines recommending treatment at CD4+ counts ≤ 500 cells/μL [10]. The post-2014 decline is consistent with patterns reported in England and Wales following similar policy transitions [11]. The steeper-than-projected decline during 2020–2021 may be associated with COVID-19-related reductions in HIV testing and clinical contact, a phenomenon documented across multiple national surveillance systems [12]. The language “may be associated with” is used deliberately, as this is an ecological analysis of aggregate data and direct causal inference cannot be established without individual-level clinical data.

The dramatic increase in the M:F notification ratio—from 0.8:1 in the late 1980s to 22.0:1 by 2024—is among the most striking findings of this analysis. This trajectory diverges sharply from global trends, where women account for approximately half of all new HIV infections worldwide [1]. In the Korean context, this shift reflects the near-disappearance of heterosexual transmission among females (31 female cases in 2024 versus 683 males) and the consolidation of MSM as the dominant transmission route [13].

The significant increase in the MSM proportion among reported sexual transmissions (slope = +1.30%/year, R^2^ = 0.804, *p* < 0.001) underscores that HIV in South Korea is now predominantly concentrated within a defined behavioral risk group. However, this finding must be interpreted in the context of the substantial non-response rate in transmission route classification (29.6% in 2024). A sensitivity analysis demonstrated that, under the assumption that non-respondents follow the same distribution as respondents (base case), the reported 63.7% MSM proportion remains the best available estimate. However, if stigma-related underreporting disproportionately affects MSM, the true proportion could be higher. Conversely, if non-response is higher among heterosexual individuals, the estimate may be an overestimate. This uncertainty should be acknowledged when interpreting the magnitude of the MSM concentration trend.

Regarding the CD4+ T-cell trend, the observed significant positive trend in late diagnosis (CD4+ < 200 cells/μL, slope = +0.0044/year, R^2^ = 0.336, *p* < 0.001) appears paradoxical given overall declining incidence and expanded ART. This finding may be explained by several mechanisms. First, the “test not performed” category remains large (particularly post-2017), and if testing is selectively performed in symptomatic or sicker patients, this introduces ascertainment bias that inflates the proportion with low CD4+ counts among those tested. Second, the MSM population—now the dominant risk group—may have differential access to or uptake of routine testing compared with other risk groups, leading to later presentation. Third, the declining overall incidence means that the absolute number of late presenters may be stable even as a proportion. These mechanisms collectively suggest that the positive CD4+ trend reflects a genuine but multifactorial public health challenge rather than a simple worsening of population-level late diagnosis.

The significant downward trend in the AIDS/HIV notification ratio (slope = −0.006/year, *p* = 0.012) is an important proxy indicator of shifting HIV natural history under Korea’s public health response. This reduction is consistent with earlier HIV diagnosis through expanded voluntary counseling and testing and more rapid linkage to ART, which delays AIDS-defining illness. Our finding that the proportional mortality rate declined from 2.31% to 0.93% (R^2^ = 0.821, *p* < 0.001) is consistent with documented survival benefits of modern ART regimens [7] and aligns with Korean-specific mortality data reported by Lee et al. [14].

The epidemiological decoupling of mortality from new infection counts—demonstrated by non-significant correlations and multiple regression (R^2^ = 0.062, *p* = 0.705)—indicates that contemporaneous notification counts no longer drive year-to-year mortality patterns. Rather, mortality is sustained by the aging prevalent cohort, characteristic of a mature epidemic under effective ART coverage [15]. The stable ARIMA-derived forecast of approximately 158 deaths per year through 2028 reflects this cohort-aging dynamic, even as incidence continues to decline.

No population-level data on PrEP uptake or PEP utilization rates are available through the KOSIS national surveillance system for the study period. PrEP has been accessible in South Korea since 2019 on a fee-based basis, and PEP has been available for longer, but national uptake data are not systematically reported. These programmatic factors may contribute to the post-2019 decline in notifications and will be important to monitor as their coverage expands toward UNAIDS 95–95–95 targets [9].

Several limitations should be acknowledged. First, all analyses were performed on pre-aggregated national statistics, precluding individual-level covariate adjustment or survival analysis. Second, transmission route non-response (29.6% in 2024) introduces potential misclassification bias, as described in Section 3.5. Third, datasets cover Korean nationals only; foreign nationals are excluded, and trends may not represent the full HIV epidemiology of the Korean resident population. Fourth, the 14-year time series for mortality and AIDS analyses limit statistical power. Fifth, causality cannot be inferred from ecological trend analyses, and ecological associations may not reflect individual-level relationships.

## 5. Conclusions

South Korea’s HIV epidemic has undergone a profound epidemiological transition over four decades of national surveillance, evolving from heterosexual-predominant early epidemic growth through a 2014 peak to a currently declining, MSM-concentrated trajectory. The statistical evidence for a biphasic incidence trend, a significant AIDS/HIV ratio decline, improving proportional mortality rates, and a widening M:F disparity is robust across multiple analytical frameworks. Time series projections suggest continued modest decline to approximately 666 new HIV notifications annually by 2028. The decoupling of mortality from incidence trends underscores the transformative impact of expanded ART coverage. However, persistently high rates of late diagnosis (CD4+ < 200 cells/μL) and the concentration of new infections among MSM underscore the continued need for targeted prevention, regular testing, expansion of PrEP access, and timely treatment linkage. Non-response bias in transmission route reporting warrants continued methodological attention in national surveillance analyses.

## Figures and Tables

**Table 1:** Annual HIV and AIDS notifications, HIV-related mortality, and cumulative case counts, South Korea, 2011–2024.

Year	HIV (*n*)	HIV Rate ^1^	AIDS (*n*)	AIDS Rate ^1^	Deaths (*n*)	Cumul. Total	Cumul. Deaths ^2^	Living PLHIV
2011	888	1.8	273	0.548	161	8541	1576	6965
2012	866	1.7	201	0.402	150	9407	1726	7681
2013	1011	2.0	192	0.382	150	10,418	1876	8542
2014	1081	2.1	265	0.522	142	11,499	2018	9481
2015	1018	2.0	202	0.396	153	12,517	2171	10,346
2016	1059	2.1	216	0.423	159	13,576	2330	11,246
2017	1008	2.0	170	0.332	171	14,584	2501	12,083
2018	988	1.9	176	0.343	135	15,572	2636	12,936
2019	1006	2.0	182	0.355	123	16,578	2759	13,819
2020	816	1.6	156	0.301	107	17,394	2866	14,528
2021	770	1.5	153	0.296	112	18,164	2978	15,186
2022	824	1.6	149	0.291	142	18,988	3120	15,868
2023	749	1.5	130	0.254	158	19,737	3278	16,459
2024	714	1.4	140	0.266	158	20,451	3436	17,015

^1^ Per 100,000 population. ^2^ Cumulative deaths = Cumulative Total − Living PLHIV. PLHIV = persons living with HIV. Source: KDCA via KOSIS.

**Table 2:** Summary of regression and correlation analyses.

Analysis	Variable(s)	Coefficient/Statistic	R^2^	*p*-Value
Linear regression	HIV notifications (1985–2024) vs. year	slope = +30.33 cases/yr	0.828	<0.001
Segmented regression	Ascending phase (1985–2014)	slope = +39.25 cases/yr	0.912	<0.001
Segmented regression	Declining phase (2014–2024)	slope = −38.76 cases/yr	0.879	<0.001
Linear regression	Male HIV notifications (1985–2024)	slope = +28.98 cases/yr	0.836	<0.001
Linear regression	Female HIV notifications (1985–2024)	slope = +1.35 cases/yr	0.552	<0.001
Linear regression	AIDS notifications (2011–2024)	slope = −8.81 cases/yr	0.729	<0.001
Linear regression	Proportional mortality rate (2011–2024)	slope = −0.105%/yr	0.821	<0.001
Linear regression	M:F ratio (1987–2024)	slope = +0.448/yr	0.600	<0.001
Linear regression	MSM proportion (1985–2024)	slope = +1.30%/yr	0.804	<0.001
Pearson correlation	HIV vs. AIDS (2011–2024)	r = 0.627	—	0.017
Spearman correlation	HIV vs. AIDS (2011–2024)	ρ = 0.785	—	<0.001
Pearson correlation	HIV vs. Deaths (2011–2024)	r = 0.191	—	0.511
Pearson correlation	AIDS vs. Deaths (2011–2024)	r = 0.243	—	0.403
Multiple regression	Deaths ~ HIV + AIDS notifications	F(2,11) = 0.361, R^2^ = 0.062	0.062	0.705
Linear regression	CD4+ < 200 proportion (1990–2024)	slope = +0.0044/yr	0.336	<0.001

**Table 3:** Time series forecast of HIV notifications, AIDS notifications, and HIV-related deaths, South Korea, 2025–2028.

Year	HIV Cases (Projected)	AIDS Cases (Projected)	Deaths (Projected)	Method
2025	702	125	158	Holt–Winters damped trend (HIV, AIDS); ARIMA(1, 1, 0) (Deaths)
2026	690	118	158	Holt–Winters damped trend (HIV, AIDS); ARIMA(1, 1, 0) (Deaths)
2027	678	111	158	Holt–Winters damped trend (HIV, AIDS); ARIMA(1, 1, 0) (Deaths)
2028	666	105	158	Holt–Winters damped trend (HIV, AIDS); ARIMA(1, 1, 0) (Deaths)

Holt–Winters = double exponential smoothing with damped additive trend, optimized smoothing parameters. Approximate 95% prediction intervals: HIV ± 8%, AIDS ± 15% of point forecast.

## Data Availability

The data analyzed in this study are publicly available as aggregate annual national statistics via the Korean Statistical Information Service (KOSIS; https://kosis.kr) and the Korea Disease Control and Prevention Agency (KDCA; https://kdca.go.kr). All analyses were performed exclusively on pre-aggregated annual national statistics; individual-level data were not accessed. The aggregated source datasets are available from the corresponding author upon reasonable request, subject to applicable data-use permissions.

## References

[1] Joint United Nations Programme on HIV/AIDS (UNAIDS) (2024). Global HIV & AIDS Statistics—Fact Sheet 2024.

[2] Maartens G., Celum C., Lewin S.R. (2014). HIV infection: Epidemiology, pathogenesis, treatment, and prevention. Lancet.

[3] Korea Disease Control and Prevention Agency (KDCA) (2025). Annual Report on the Notified HIV/AIDS in Korea.

[4] Kim J.M., Cho G.J., Hong S.K., Chang K.H., Chung J.S., Choi Y.H., Song Y.G., Huh A., Yeom J.S., Lee K.S. (2003). Epidemiology and clinical features of HIV infection/AIDS in Korea. Yonsei Med. J..

[5] Choi Y., Choi B.Y., Kim S.M., Kim S.I., Kim J., Choi J.Y., Kim S.W., Song J.Y., Kim Y.J., Park D.W. (2019). Epidemiological characteristics of HIV-infected Koreans: Korea HIV/AIDS Cohort Study. Epidemiol. Health.

[6] Choi B.Y., Choi J.Y., Han S.H., Kim S.I., Kee M.-K., Kim M.J., Kim S.-W., Kim S.S., Kim Y.-M., Ku N.S. (2018). Korea HIV/AIDS Cohort Study: Study design and baseline characteristics. Epidemiol. Health.

[7] Samji H., Cescon A., Hogg R.S., Modur S.P., Althoff K.N., Buchacz K., Burchell A.N., Cohen M., Gebo K.A., Gill M.J. (2013). Closing the gap: Increases in life expectancy among treated HIV-positive individuals in the United States and Canada. PLoS ONE.

[8] Nakagawa F., Lodwick R.K., Smith C.J., Smith R., Cambiano V., Lundgren J.D., Delpech V., Phillips A.N. (2012). Projected life expectancy of people with HIV according to timing of diagnosis. AIDS.

[9] Joint United Nations Programme on HIV/AIDS (UNAIDS) (2015). Understanding Fast-Track: Accelerating Action to End the AIDS Epidemic by 2030.

[10] World Health Organization (2013). Consolidated Guidelines on the Use of Antiretroviral Drugs for Treating and Preventing HIV Infection: Recommendations for a Public Health Approach.

[11] Birrell P.J., Gill O.N., Delpech V.C., Brown A.E., Desai S., Chadborn T.R., Rice B.D., De Angelis D. (2013). HIV incidence in men who have sex with men in England and Wales 2001–10: A nationwide population study. Lancet Infect. Dis..

[12] Kee M.K., Lee J.H., Kim E.J., Lee J., Nam J.G., Yoo B.H., Kim S.S. (2009). Improvement in survival among HIV-infected individuals in the Republic of Korea: Need for an early HIV diagnosis. BMC Infect. Dis..

[13] Kee M., Lee K.H., Lee S.Y., Kang C., Chu C. (2014). Trends and Characteristics of HIV Infection among Suspected Tuberculosis Cases in Public Health Centers in Korea: 2001–2013. Osong Public Health Res. Perspect..

[14] Lee S.H., Kim K.H., Lee S.G., Chen D.H., Jung D.S., Moon C.S., Park J.Y., Chung J.S., Kwak I.S., Cho G.J. (2013). Trends of mortality and cause of death among HIV-infected patients in Korea, 1990–2011. J. Korean Med. Sci..

[15] Kim Y., Kim S.W., Chang H.H., Kwon K.T., Bae S., Hwang S. (2020). Trends of cause of death among human immunodeficiency virus patients and the impact of low CD4 counts on diagnosis to death. J. Korean Med. Sci..

